# Comparison of the 7th and 8th editions of the American joint committee on cancer TNM classification for patients with stage III gastric cancer

**DOI:** 10.18632/oncotarget.18375

**Published:** 2017-06-06

**Authors:** Jun Lu, Chao-Hui Zheng, Long-Long Cao, Ping Li, Jian-Wei Xie, Jia-Bin Wang, Jian-Xian Lin, Qi-Yue Chen, Mi Lin, Ru-Hong Tu, Chang-Ming Huang

**Affiliations:** ^1^ Department of Gastric Surgery, Fujian Medical University Union Hospital, Fuzhou, China; ^2^ Department of General Surgery, Fujian Medical University Union Hospital, Fuzhou, China; ^3^ Key Laboratory of Ministry of Education of Gastrointestinal Cancer, Fujian Medical University, Fuzhou, China; ^4^ Fujian Key Laboratory of Tumor Microbiology, Fujian Medical University, Fuzhou, China

**Keywords:** gastric cancer, TNM classification, prognosis, 8th edition

## Abstract

**Background and Objectives:**

The eighth TNM edition for gastric cancer was released in 2016 and included major revisions, especially of stage III. The purpose of this study was to evaluate the prognostic value of the new AJCC TNM classification in comparison with the 7th edition for stage III gastric cancer.

**Methods:**

Clinical and histopathological data on 1,496 patients operated on for stage III GC according to the seventh edition between 2005 and 2013 were analyzed and compared using 7th and 8th classifications. The 2 systems were compared in terms of prognostic performance.

**Results:**

The stage shifted for 650 (43.45%) patients: from IIIA to IIIB (2 patient, 0.13%), from IIIB to IIIA (214 patients, 14.30%), from IIIB to IIIC (99 patients, 6.62%), and from IIIC to IIIB (335 patients, 22.39%). Cox regression multivariate analysis showed both the 8th and 7th TNM classification were independent prognostic factors. The 8th edition system had higher linear trend and likelihood ratio χ^2^ scores, and smaller AIC values compared with those for the 7th edition. However, the performance of the eighth edition did not reveal significant improvement compared to the seventh edition (c-index 0.625 vs. c-index 0.616, p=0.085).

**Conclusion:**

The eighth TNM edition may not provide significantly better accuracy in predicting the prognosis of stage III GC. However, to confirm our findings, further studies are warranted.

## INTRODUCTION

The AJCC TNM system is recognized as the most well-established and well-recognized malignant tumor staging system worldwide. Over the past decades, the AJCC TNM staging system has been revised continuously, and the most recent eighth edition of the TNM classification published in 2016 replaced the seventh edition from 2009 [1]. Changes to the latest classification of gastric cancer are mainly based on data analyses from the US and Japan.

**Figure 1:**
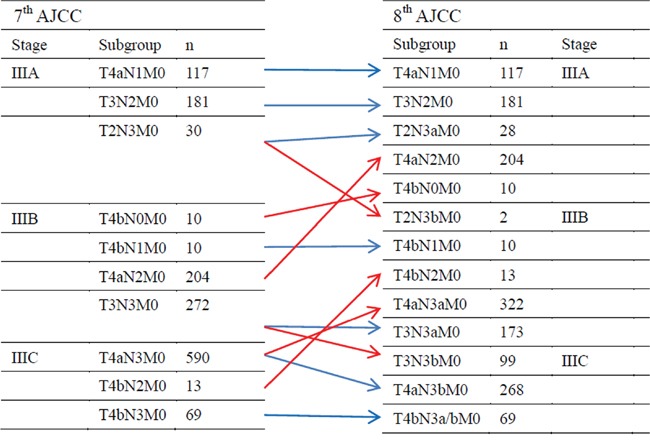
AJCC stage and TNM subgroup distributions of the patients according to the seventh and eighth editions of the TNM classification

The seventh edition N3 stage was divided into N3a (7–15 positive regional lymph nodes) and N3b (>15 positive regional lymph nodes). However, in the 7th edition, the N3 sub-classification (N3a and N3b) do not differ with regards to the final pathologic stage [2]. Recently, the AJCC published the eighth edition of the TNM classification, and several changes to the 8th edition of the AJCC staging system for gastric cancer have been proposed (Supplementary Table 1) [1]. A key change adopted in new eighth edition details pN3 as pN3a and pN3b in the final pathologic stage. Thus, a comparison of stage distributions between old and new TNM classifications shows that stages I and II did not change except for T1N3bM0 (changing from IIB in the 7th ed. to IIIB in the 8th ed.). The main modification involved a major change to stage III. T2N3bM0 tumors were upstaged from stage IIIA to IIIB, and T3N3bM0 tumors were upstaged from IIIB to IIIC. In addition, T4bN0M0 and T4aN2M0 tumors were downstaged from IIIB to IIIA. Finally, T4aN3aM0 and T4bN2M0 tumors were downstaged from IIIC to IIIB (Figure 1). As stated above, the most important change made to the 8th edition concerns stage III of gastric cancer. Therefore, in the present study, we mainly evaluated classification changes made in regards to stage III gastric cancer.

To our knowledge, this is the first study to examine GC of the 8th TNM system. The objective of the present study was to evaluate the validity of the proposed 8th edition AJCC system and to identify the optimal TNM classification for stage III gastric cancer based on the prospectively collected database from a large specialized eastern center.

## RESULTS

### Patients characteristics

Data on 1,496 consecutive patients were analyzed. In total, 1,101 (73.6%) of the patients were male and 395 (26.4%) were female with a median age of 62 years (range, 16–101 years). The mean number of dissected LNs was 34.5 (range 5–108). The patient and histopathological characteristics are shown in Table 1.

**Table 1:** Univariate analysis of clinicopathologic factors for 5-year survival rate

Factor	Number	5-year survival rate (%)	*P* value
Age			<0.001
<65	896	37.3	
≥65	600	27.0	
Gender			0.484
Male	1101	34.6	
Female	395	28.8	
Tumor location			0.028
Upper	524	30.2	
Middle	384	32.0	
Lower	588	36.5	
Tumor size(cm)			<0.001
<5.0	399	53.2	
≥5.0	1097	26.8	
Histological type, n(%)			0.181
Differentiated	557	36.7	
Undifferentiated	939	33.9	
N stage (7^th^ AJCC)			<0.001
N0	10	51.4	
N1	127	53.8	
N2	398	46.1	
N3	961	27.4	
N stage (8^th^ AJCC)			<0.001
N0	10	51.4	
N1	127	53.8	
N2	398	46.1	
N3a	561	30.5	
N3b	400	15.4	
TNM stage (7^th^ AJCC)			<0.001
IIIA	331	53.9	
IIIB	493	38.1	
IIIC	672	19.8	
TNM stage (8^th^ AJCC)			<0.001
IIIA	550	49.2	
IIIB	529	30.0	
IIIC	417	15.4	

### Seventh and eighth editions of the AJCC TNM classification

Classifying according to the 2 editions revealed that 961 patients with N3 tumors were divided into 561 patients with N3a (58.4%) and 400 patients with N3b (41.6%). The AJCC stage distributions according to the seventh and eighth editions of the TNM classification are shown in Figure 1. 7th IIIA stage differentiate into 8th IIIA stage and 8th IIIB stage; 7th IIIB stage differentiate into 8th IIIA stage, 8th IIIB stage and 8th IIIC stage; 7th IIIC stage differentiate into 8th IIIB stage and 8th IIIC stage. Our comparison of the 2 classifications revealed that AJCC III stage tumors changed in 650 (43.45%), which was defined as the staging shift; 846 (56.55%) patients were not changed in the 8th edition, which was defined as the staging stable. In detail, patients were reclassified from AJCC stage IIIA to IIIB (2 patient, 0.13%), from IIIB to IIIA (214 patients, 14.30%), from IIIB to IIIC (99 patients, 6.62%), and from IIIC to IIIB (335 patients, 22.39%).

### Survival differences between the staging shift and staging stable patients

**Figure 2:**
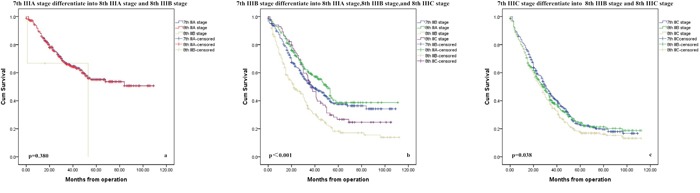
Survival curves by subgroups according to different subgroup of 7th AJCC classification

**Figure 3:**
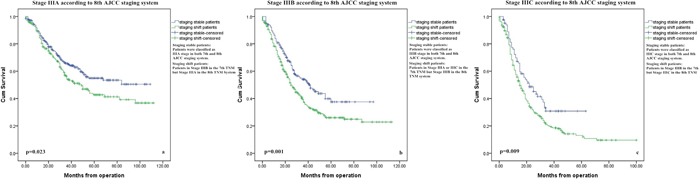
Survival curves for patients with stage III gastric cancer according to their subgroups

Figure 2a-2c presents the overall survival curves of the patients according to the classification in the 7th and 8th edition of the AJCC staging system. IIIA stage did not show statistical differences because too few patients changed to new stage in the 8th edition (Figure 2a, p=0.380). However, the survival curves for staging shift patients versus staging stable patients of stage IIIB/IIIC subgroups according to the classification in the 7th edition AJCC system were significantly different (Figure 2b-2c, p<0.05). Significant differences between survival curves of the staging shift and staging stable patients were also observed in 8th AJCC classifications (Figure 3a-3c, p<0.05).

### Univariate and multivariate survival analysis

**Figure 4:**
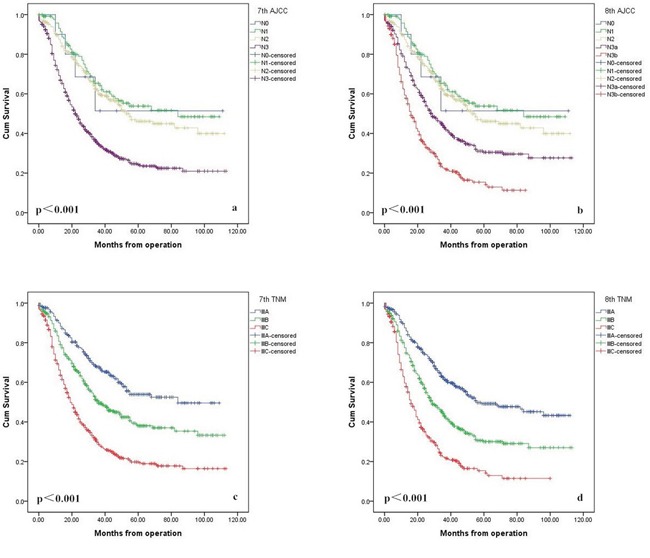
Comparison of survival curves according to N stage between the 7th **(a)** and 8th edition **(b)**, and TNM classifications between the 7th **(c)** and 8th edition **(d)**.

In univariate analysis, age, tumor size, tumor location, N stage (7th AJCC), N stage (8th AJCC), TNM stage (7th AJCC), and TNM stage (8th AJCC) were significantly correlated with patients’ 5-year OS (Table 1). We thus performed multivariate Cox proportional hazard model analysis for factors that had significant correlation with OS. The result showed that the N stage (7th AJCC), N stage (8th AJCC), TNM stage (7th AJCC), and TNM stage (8th AJCC) were the independent prognostic factors. (Table 2). Figure 4 shows the patient stage-specific survival curves according to the 7th and 8th classifications.

**Table 2:** Multivariate analysis with Cox proportional hazard model for prognostic factors

Factor	Hazard ratio (HR)	95% CI	P value
7th N stage (AJCC)	1.224	0.482-0.867	0.005
8th N stage (AJCC)	1.657	1.218-2.255	0.004
7th TNM stage (AJCC)	1.469	1.260-1.714	<0.001
8th TNM stage (AJCC)	1.735	1.271-2.367	<0.001

### Comparisons between the two prognostic classification systems

The performance of the 7th and 8th edition staging system assessed by the C-index, AIC, likelihood ratio χ^2^ score, and linear trend χ^2^ score is presented in Table 3. A statistical assessment of the prognostic performance of the 2 AJCC classification editions based on the c-index reveals a value of 0.616 (95% CI, 0.597-0.635) for the 7th edition and a value of 0.625 (95%CI, 0.604-0.642) for the 8th edition, however, the difference was not statistically significant (p=0.085).

**Table 3:** Comparison of the performance of the 7^th^ and the 8^th^ edition AJCC TNM staging system

	Concordance indices		AIC	Likelihood ratio χ^2^	Linear trend χ^2^
	C-index	Bootstrap 95% CI			
7^th^ AJCC system	0.616	0.597 to 0.635	1568.91	6861.243	5817.383
8^th^ AJCC system	0.625	0.604 to 0.642	1559.39	6935.005	5882.391

## DISCUSSION

Gastric cancer (GC) is the second leading cause of cancer-related death, with the highest mortality rates found in East Asia, including Japan, Korea, and China [3]. Surgical resection remains the main form of treatment. However, despite advances made in treatment strategies over past decades, the prognosis for stage III gastric cancer is still poor. In China, where gastric cancer is endemic, the majority of patients are diagnosed at middle or late stages, reflecting poor overall survival rates [4]. Therefore, we finally focused on stage III, which represents approximately 50% of all entire gastric cancer cases diagnosed in China.

Several important changes were made to the recently modified 8th edition of the TNM staging system of gastric cancers released in 2016 from the 7th edition. Changes made to the TNM classification and AJCC tumor stages were based on survival analyses performed for gastric cancer listed the NCDB (U.S.) and Shizuoka Cancer Center (Japan) dataset. The eighth edition of the TNM classification system includes substantial changes for gastric cancer, providing more comprehensive tools (cTNM, ypTNM, and pTNM) for the stage grouping of gastric cancer patients under different circumstances that may influence treatments and that may serve as the basis of future clinical studies [5]. In this paper, we mainly discuss changes made to the pTNM classification. The introduction of several new subgroups and sub-stages resulted in the creation of a complex and confusing classification for daily clinical use (Figure 1 and Supplementary Table 1).

Concerning the N category in the 8th edition, the main change made to this category involved splitting N3 staging into N3a (7–15 positive LNs) and N3b (more than 15 LNs nodes). Although the seventh edition N3 classification was sub-classified as N3a and N3b, each subgroup was not an individual determinant of the final TNM stage, which may cause serious problems in underestimating GC severity levels. There is now enough proof of the limitations of the 7th AJCC N3 classification, and the need for N classification modifications was raised by various investigators prior to the introduction of the 8th edition AJCC TNM classification [6–10]. Therefore, the 8th edition of the TNM classification adopted numeric classifications for N3 classification, and it was divided into 2 subgroups in the final TNM stage. The involvement of ≥16 lymph nodes (N3b) was associated with worse outcomes than cases involving 7-15 positive nodes (N3a) according to a series from Italy [8], and similar results were also found through 2 large Korean studies [10–11]. Our data also confirm that N3a and N3b may represent diseases of differing severity, and the 5-year survival rate of patients according to the eighth edition N3a classification is also significantly better than that of patients with N3b stage tumors. Therefore, it appears reasonable to revise the seventh edition pN3(a/b) to a different pN classification even if an analysis of T1N3b and T2N3b categories was not possible due to an insufficient number of patients.

Next, we compared the IIIA, IIIB, and IIIC tumor staging guidelines of both editions. T4aN2 and T4bN0 are now classified as stage IIIA, and T4bN2 is now classified as stage IIIB. In our series, partial cases of stage IIIB (T4aN2 and T4bN0) and IIIC (T4bN2) diseases in the 7th edition system were downstaged to IIIA and IIIB in the 8th edition AJCC. Overall, down-staging was observed in 36.7% of stage III cases, whereas 6.8% of stage III cases were up-staged. However, when Marrelli et al. [8] compared the 7th system with the 6th edition, they found down-staging in 10.4% of cases and up-staging in 27.2% cases.

Staging is a key facet of cancer treatment. The accurate staging of cancer patients reveals the progression of a disease, the risk of recurrence and overall survival determinations, which have a significant influence on treatment decisions and which allow one to draw comparisons between patient cohorts across institutions and countries. Recently, International Gastric Cancer Association (IGCA), which is an academic group having nearly 1500 members from 57 countries, proposed a new stage grouping based on a large, worldwide data collection. They established a new evidence-based classification with better stratification than 7^th^ AJCC [12]. Wang et al [11] found that the 7th edition TNM system performs better than the 6th edition in several aspects. A study by Warneke et al [13] concluded that the 7th AJCC classification has become more complex without improving predictability for overall survival in a Western population. Therefore, the authors believed that a simplification of the staging systems for gastric cancer seems justified. Another eastern study found that some subgroups of the seventh edition TNM classification did not demonstrate significantly different survival rates [14]. The 8th edition of the TNM classification attempts to show significant differences in stage III disease survival rates by using a more ideal structure relative to that of the 7th edition staging system. According to our survival analysis, the 8th TNM edition is more accurate in predicting stage III gastric cancer patients’ prognoses than the 7th edition. However, similar as reported in other malignancy [15, 16], it is worth noting that the c-index in this research is less than 0.75, and potentially because stage III GC includes an extremely heterogeneous group of diseases, thus potentially prohibiting the creation of any meaningful stage grouping based solely on local tumor growth and nodal spread patterns. Other variables will show to significantly influence patient survival rates such as histological and molecule phenotypes. Progress will be achieved by combining the TNM classification system with molecular tools [17].

This study presents several limitations. First, this study was retrospective despite being performed based on a prospectively collected database. It was performed based on data from specialized centers with standardized lymphadenectomy and node retrieval capabilities, and this must be considered when comparing results with other cases. Second, we did not analyze the effects of postoperative chemotherapy procedures on prognoses. Third, the validation of this proposed classification system in another cohort, particularly in a Western population, should be performed. Fourth, the median follow-up period used was only 53 months, which maybe not be a long enough period to support definite conclusions. To address these limitations, our results should be validated for different series based on large sample sizes and sufficient follow-up periods.

## CONCLUSIONS

In summary, we first validate the superior prognostic and discriminating value of the 8th edition AJCC classification for stage III gastric cancer patients. However, novel prognostic biomarkers are urgently needed. We believe that progression of precise stratification tools for the prediction of patient prognoses will be achieved by combining the TNM subgroup classification system with molecular tools, in the near future.

## MATERIALS AND METHODS

### Patients

This study was designed as a retrospective analysis based on prospectively collected data. Between 2005 and 2013, 1,496 patients underwent curative resection with D2 lymphadenectomy for stage III gastric cancer according the seventh edition of the AJCC TNM classification [2] at the Department of Surgery of Fujian Medical University Union Hospital (Table 1). The surgical strategy was according to the Japanese Gastric Cancer Treatment Guidelines. The data on these patients included information on demographic parameters, histopathologic tumor characteristics, and survival rates. We excluded the following patients from the study: (1) patients with pathological I or II stage conditions, (2) patients undergoing palliative surgery, (3) patients with distant metastasis, and (4) patients with synchronous malignancies. (5) We excluded patients who had undergone neoadjuvant chemotherapy because in the 8th edition AJCC cancer staging manual, a special postneoadjuvant therapy stage (ypTNM) grouping system had been provided based on United States National Cancer Data Base (NCDB). The flowchart of the patient selection process was shown in Supplementary Figure 1. The 8th TNM classification's application was simulated in these cases and was compared on a case-by-case basis with the 7th edition of TNM staging.

All operations were performed by experienced surgeons who had experience of more than 300 cases of gastrectomy before study start [18]. Adjuvant chemotherapy with 5-fluorouracil (5-FU)-based regimens (mostly 5-FU with cisplatin) was recommended to the eligible patients. Postoperatively, patients were examined during follow-up visits every 3 months for the first 2 years and every 6 months thereafter. At each follow-up control, carcinoembryonic antigen and carbohydrate antigen 19-9 levels were measured. Thoracicoabdominal and pelvic computed tomographic scanning or abdominal ultrasonography was performed alternately every 3-6 months. Gastroscopy was performed yearly. 1,349 patients (90.2%) were followed up with, and the median follow-up was 53 months (range 2-115).

This study was approved by the Institutional Review Board of the Ethical Committee of Fujian Medical University Union Hospital.

### Definitions of the eighth edition TNM classification

For the eighth edition pTNM classification, definitions of T and N classifications were not changed, and only the final staging assignment of the pN3 classification was changed. The seventh edition pN3 classification was divided into pN3a and pN3b classifications in the eighth edition, and the seventh edition pT4aN2 and T4bN0 classifications were reclassified as stage IIIA in the eighth edition [1]. Supplementary Table 1 shows detailed classifications based on the seventh and eighth editions of the AJCC TNM classification.

### Statistical analysis

All data were analyzed by statistical analysis program package (SPSS 17.0, SPSS Inc., Chicago, IL, USA), and the statistical software“R” (version 2.11.1, the R Foundation for statistical computing). Survival time was calculated from the day of surgical resection, and the day of death or last follow-up was considered as endpoint. Overall survival (OS) was calculated using the Kaplan-Meier method, and the log-rank test was employed to determine the significance. The likelihood ratio χ^2^ test related to the Cox regression model was used to measure homogeneity. The discriminatory ability and monotonicity of gradient assessments were measured with the linear trend χ^2^ test. To assess potential bias in comparing prognostic systems with different numbers of stages, the Akaike information criterion (AIC) within the Cox proportional-hazard regression model was used. [19]. The predictive accuracy of the model was also evaluated by the concordance index (C-index) [20], which can range from perfect concordance (1.0) to perfect discordance (0.0), the corresponding confidence interval (CI) were obtained by bootstrapping, as previously described [16]. p values for the C-index were computed by assuming asymptotic normality [15]. All statistical tests were performed 2-sided, and a p<0.05 was considered as statistically significant.

## SUPPLEMENTARY MATERIALS FIGURES AND TABLES


