# Supplementary material for: Comparison of the 7th and 8th editions of the American joint committee on cancer TNM classification for patients with stage III gastric cancer
Source: Oncotarget. 2017 Jun 6;8(48):83555–62. doi: 10.18632/oncotarget.18375 (PMC5663535; doi:10.18632/oncotarget.18375)
Supplement: Supplementary file 1 [file oncotarget-08-83555-s001.pdf]

Comparison of the 7th and 8th editions of the American joint committee on cancer TNM classification for patients with stage III gastric cancer

SUPPLEMENTARY FIGURE AND TABLE

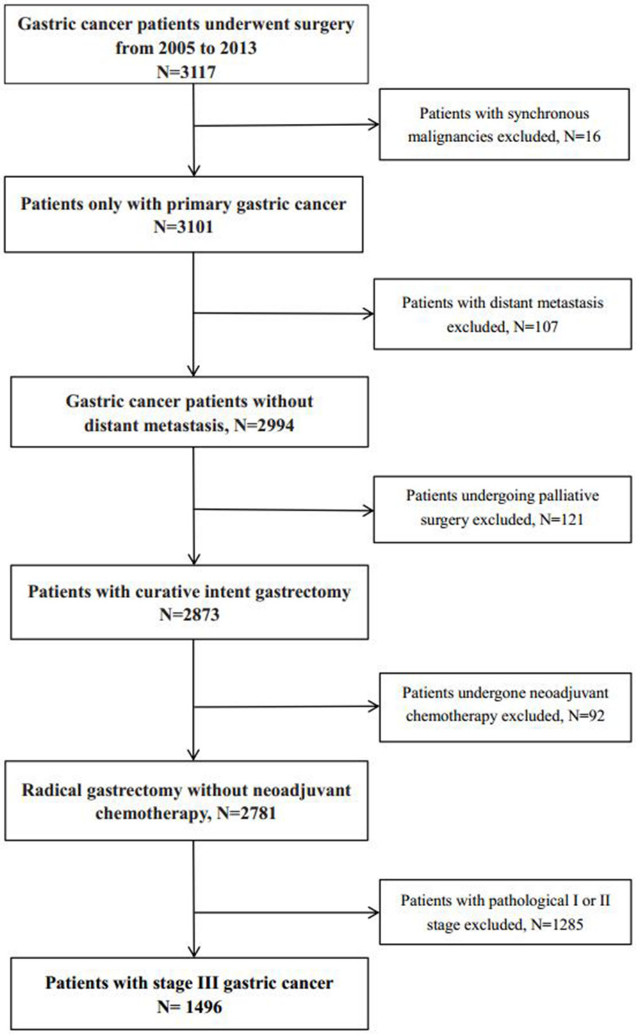

Supplementary Figure 1: Study cohort selection flowchart.

Supplementary Table 1: Differences in the TNM Classification Between the Seventh, and Eighth Editions

AJCC seventh edition	N0	N1(1-2)	N2(3-6)	N3(7-)	Any N, M1
T1	IA	IB	IIA	IIB	IV
T2	IB	IIA	IIB	IIIA	
T3	IIA	IIB	IIIA	IIIB	
T4a	IIB	IIIA	IIIB	IIIC	
T4b	IIIB	IIIB			
Any T, M1					

AJCC eighth edition	N0	N1(1-2)	N2(3-6)	N3a(7-15)	N3b(16-)	Any N, M1
T1	IA	IB	IIA	IIB	IIIB	IV
T2	IB	IIA	IIB	IIIA		
T3	IIA	IIB	IIIA	IIIB	IIIC	
T4a	IIB	IIIA	IIIA			
T4b	IIIA	IIIB	IIIB			
Any T, M1						
